# Emissions from Hydrogen Peroxide Disinfection and Their Interaction with Mask Surfaces

**DOI:** 10.1021/acsengineeringau.3c00036

**Published:** 2024-01-09

**Authors:** Pearl Abue, Nirvan Bhattacharyya, Mengjia Tang, Leif G. Jahn, Daniel Blomdahl, David T. Allen, Richard L. Corsi, Atila Novoselac, Pawel K. Mistzal, Lea Hildebrandt Ruiz

**Affiliations:** † McKetta Department of Chemical Engineering, The University of Texas at Austin, Austin, Texas 78712, United States; ‡ Department of Civil, Architectural, and Environmental Engineering, The University of Texas at Austin, Austin, Texas 78712, United States; § College of Engineering, University of California, Davis, Davis, California 95616, United States

**Keywords:** Hydrogen peroxide, disinfection, COVID-19, face masks, indoor air quality

## Abstract

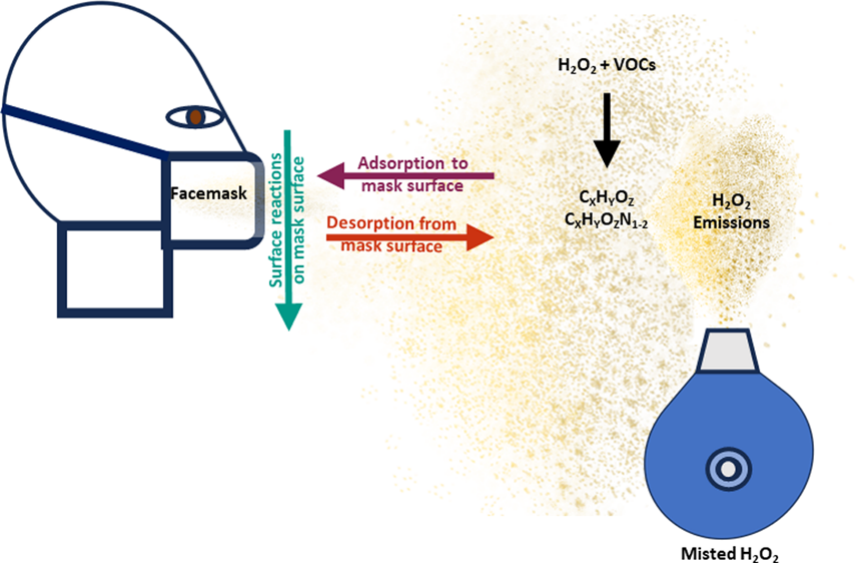

A rise in the disinfection of spaces occurred as a result of the COVID-19 pandemic as well as an increase in people wearing facial coverings. Hydrogen peroxide was among the recommended disinfectants for use against the virus. Previous studies have investigated the emissions of hydrogen peroxide associated with the disinfection of spaces and masks; however, those studies did not focus on the emitted byproducts from these processes. Here, we simulate the disinfection of an indoor space with H_2_O_2_ while a person wearing a face mask is present in the space by using an environmental chamber with a thermal manikin wearing a face mask over its breathing zone. We injected hydrogen peroxide to disinfect the space and utilized a chemical ionization mass spectrometer (CIMS) to measure the primary disinfectant (H_2_O_2_) and a Vocus proton transfer reaction time-of-flight mass spectrometer (Vocus PTR-ToF-MS) to measure the byproducts from disinfection, comparing concentrations inside the chamber and behind the mask. Concentrations of the primary disinfectant and the byproducts inside the chamber and behind the mask remained elevated above background levels for 2–4 h after disinfection, indicating the possibility of extended exposure, especially when continuing to wear the mask. Overall, our results point toward the time-dependent impact of masks on concentrations of disinfectants and their byproducts and a need for regular mask change following exposure to high concentrations of chemical compounds.

## 1 Introduction

The national human activity pattern survey (NHAPS) showed that in the United States, humans spend approximately 90% of their time indoors.^1^ Indoor spaces are disinfected regularly to achieve a desired degree of perceived cleanliness^2^ and to prevent the spread of diseases through the deactivation of disease-causing microorganisms.^2^ Chemical disinfectants containing bleach, hydrogen peroxide, benzalkonium chlorides, and other chemicals are commonly utilized for disinfecting air and surfaces in homes, schools, and hospital wards.^3−5^

SARS-CoV-2 (coronavirus, COVID-19) was declared a global pandemic by the World Health Organization (WHO) on the 11th of March, 2020.^6^ Multiple measures were recommended to prevent and control the spread of the virus. One of those measures was the disinfection of rooms and spaces to deactivate the virus,^6^ which would otherwise have a median half-life of 5.6 h on stainless steel surfaces and 6.8 h on plastic surfaces.^7^ Several disinfectants were recommended for disinfecting surfaces to prevent the spread of the virus including bleach, hydrogen peroxide, and others. Hydrogen peroxide (H_2_O_2_) has long been used as a highly effective disinfectant toward vegetative bacteria, fungi, and viruses^3,8,9^ and has been used in both the traditional liquid application and its vaporous form via no-touch devices for efficient disinfection by the pharmaceutical industry, food industry, and hospitals.^3^ During the pandemic, the use of vaporous hydrogen peroxide was expanded to include disinfecting N95 face masks for reuse during mask shortages^10,11^ and various multioccupant spaces to prevent the spread of COVID-19.^6,12^

In addition to disinfection, temporarily wearing a facial covering to prevent the spread of respiratory particles when interacting with people was recommended.^6^ Various kinds of facial coverings were worn, such as N95 (including respirators), KN95, surgical, and cloth masks. Masks were recommended for one-time use or reuse after careful decontamination.^13−15^ Prior to the pandemic, wearing masks had been prevalent in some communities as a precaution against the spread of respiratory diseases or poor air quality.^16−18^ However, the need to wear masks to prevent the spread of COVID-19 brought about an increase in mask wearing and led to the widespread use of masks in communities and during activities where it had previously been uncommon.^19,20^

Hydrogen peroxide as a disinfectant has been studied in multiple contexts prior to the pandemic. One of which is its relevance to atmospheric chemistry as a major source of the hydroxyl (OH) radical when photolyzed.^21−23^ OH radicals can oxidize volatile organice compounds (VOCs) in the gas phase, and H_2_O_2_ can react in the aqueous phase to form byproducts.^21,22,24^ Models have been used to estimate emissions of methanol, formic acid, and acrolein in a room following hydrogen peroxide cleaning and predicted increased radical concentrations following cleaning.^21^ In one of the few studies characterizing hydrogen peroxide, its associated emissions and their interactions with surfaces, Poppendieck et al.^24^ studied the removal of vaporous hydrogen peroxide (VHP) to indoor surfaces and the associated emissions when used as an indoor disinfectant. They found that in some materials, organic emissions following disinfection by VHP were greater than the organic emissions following disinfection with ozone. In another study, after exposing N95 masks to 3% and 50% vaporized hydrogen peroxide, 2.4–14.5 ppm of H_2_O_2_ was observed after 30 min of aeration, concentrations above the permissible 1 ppm total weighted average (TWA) after 2–5 h of aeration.^25^ However, the byproducts were not discussed in this study. Despite multiple studies addressing the emission of hydrogen peroxide from cleaning, its removal to surfaces, and models estimating the emissions of some byproducts, additional work is necessary to characterize the array of byproducts from the use of hydrogen peroxide and the interaction of this disinfectant with masks worn during and after its use. In this work, we quantify emissions of the primary disinfectant, hydrogen peroxide, and the associated byproducts formed and emitted following the injection of a 3% hydrogen peroxide solution using a humidifier. We examine the effect of wearing a mask designed for protection against aerosols on exposure to gases when the mask is worn during and following the disinfection of an indoor space. Finally, we examine the impact of mask humidification from breathing on the concentrations behind the mask.

## 2 Experimental Section

### 2.1 Environmental Chamber Setup

Chamber experiments were conducted in a 67 m^3^ stainless steel environmental chamber located at the J. J. Pickle Research Campus at the University of Texas at Austin, which is described briefly here and in more detail elsewhere.^26,27^ The environmental chamber, which is operated as a well-mixed chamber,^27,28^ is fitted with an air handling unit (AHU) for air filtration and ventilation. For the experiments discussed here, it was operated at an air exchange rate (AER) of 2.3 h^–1^, which falls within typical classroom ventilation rates of 1.9–2.4 h^–1^.^29^ Although higher AERs were advised during the pandemic to improve ventilation and reduce exposure to the virus, these recommendations were often not implemented in school classrooms because of technological constraints and administrative challenges, making the AER used here still representative of typical classroom ventilation.^30,31^ Temperature and relative humidity (RH) in the chamber were at an average of 22.7 ± 1.7 °C and 41.3 ± 14.4%, respectively, for the duration of the experiments. To simulate a mask-wearing human, a thermal manikin, which was not heated during these experiments, was seated in the chamber, and an unused surgical or KN95 mask was placed over its nose and mouth (breathing region) prior to the start of every experiment. Manikin breathing was simulated by inhaling chamber air at 0.5 L with 12 breaths per minute, and exhalation only occurred while the mask was being humidified before the start of the disinfectant injection to avoid dilution of the chamber air during the experiment. Humidification of the mask was achieved by exhaling humidified air (>95% RH) at 37 °C onto the mask at 12 breaths per minute, leading to an exhalation rate of 6 L min^–1^. A detailed description of the humidification process can be found in the Supporting Information (SI), section SI.1. After humidification, the KN95 mask typically weighed approximately 1 g more than the dry KN95 mask, indicating that 1 g of water had adsorbed onto its surface. Also present in the chamber were painted wall boards and six tables.

Commercial 3% hydrogen peroxide (H_2_O_2_) disinfectant solution (the primary disinfectant) was injected at a constant rate into the room using an ultrasonic adiabatic humidifier (Holmes) on the “High” setting at 0.25 L h^–1^ for 2–3 h to achieve a targeted concentration of 1000 ppb. The target concentration for these experiments encompasses the 600 ppb concentrations measured when manufacturer instructions for the use of hydrogen peroxide are followed^21^ and the 1500 ppb concentrations achieved during spray tests conducted in our chamber. Multiple sampling points were placed within the chamber for sampling concentrations behind the mask (referred to as “behind mask”), inside the chamber (referred to as “inside chamber”), the inlet air into the chamber, and the outlet air from the chamber. Here, we present the data from the sampling points behind the mask and inside the chamber (located 20 cm from the mask) to focus on how concentrations are affected by the mask barrier. Instruments switched sampling locations every 5 min, and each location was sampled once every 20–30 min. A more detailed description of the sampling locations and chamber setup can be found elsewhere.^32^ The chamber temperature was not observed to change substantially over the course of an experiment, and the chamber RH was observed to increase by 10–20% during injection with no substantial change recorded during mask humidification. These increases in RH are unsurprising based on the proportion of water in the hydrogen peroxide solution and may increase the thickness of adsorbed water layers on chamber surfaces.

The instruments used for sampling during this work include a Vocus 2R proton transfer reaction time-of-flight mass spectrometer (Vocus PTR-ToF-MS)^33^ and a chemical ionization mass spectrometer operating in iodide mode (I^–^ CIMS).^34^ Both instruments sampled solely in the gas phase with the I^–^ CIMS measuring H_2_O_2_ concentrations and Vocus PTRMS measuring all other gas-phase organic compounds reported here. In order to calibrate the I^–^ CIMS for hydrogen peroxide, H_2_O_2_ was injected into a 10 m^3^ Teflon environmental chamber by bubbling clean, dry air at 2 L min^–1^ through a 30% hydrogen peroxide solution. Estimates for H_2_O_2_ concentrations during calibration were based on interference in the 254 nm photometric ozone monitor and the relative ratio of ozone and hydrogen peroxide absorption cross sections at 254 nm. More details on the calibration can be found in the SI. The Vocus PTR-ToF-MS was operated with an ion molecular reactor pressure of 2.3 mbar, a 15 sccm flow rate from the water reservoir (H_3_O^+^ source), and IMR front and back voltages of 650 and 25 V, respectively, for an E/N ratio of 150 Td. Vocus calibrations were performed daily using a standard gas mixture, and species not in this mixture were calibrated by their proton transfer reaction rate.^35^ Analytes were assumed to be detected primarily as a protonated [M]H^+^ species, and the automated PTRwid package^36^ was utilized for peak fitting and assignment of the data discussed here. Although we expected most detected species to be protonated [M]H^+^ species, the possible formation of water clusters [M]H_3_O^+^ and fragmentation products adds a layer of uncertainty to peak identification.^33,35,36^ Typically, either the I^–^ CIMS or the Vocus PTR-ToF-MS was used in each experiment, and when available, both instruments were used.

Table 1 contains details of the experiments discussed here and the instrumentation used in each experiment. In each experiment, we fitted the thermal manikin with either an unused surgical mask (BYD Electronics) or an unused KN95 mask (Kingfa), both of which are commonly used mask types. Masks were either dry or humidified to simulate a mask surface humidified by breathing. We note the absence of the complex chemical composition of human breath here, as lab air passed through a HEPA (high-efficiency particulate air) filter and humidified air was exhaled onto the mask.

**Table 1:** Experimental Conditions

#	mask	humidified mask?	instrumentation	peak H_2_O_2_ observed inside chamber (ppb)	peak H_2_O_2_ observed behind mask (ppb)	H_2_O_2_ decay rate inside chamber (h^–1^)	H_2_O_2_ decay rate behind mask (h^–1^)	chamber air change rate (h^–1^)
1	KN95	no	CIMS, Vocus	2827	2379	0.49	0.47	2.3
2	surgical	yes	CIMS	511	176	0.48	0.17	2.3
3	KN95	yes	Vocus	-	-	-	-	2.3
4	KN95	yes	Vocus	-	-	-	-	2.3
5	surgical	no	CIMS	2259	801	0.50	0.15	2.3

Organic compounds observed during these experiments are grouped into families according to their chemical formula, either oxygenated (C_2–10_H_*Y*_O_1–4_) or nitrogen-containing (C_2–10_H_*Y*_O_1–4_N_1–2_) compounds. Details of the calculations of the decay rates shown in Table 1 are discussed in section 2.2.

### 2.2 Decay Rate Calculations and Net Source–Sink Estimation

Decay rates were calculated from the first-order decay rate (eq 1) to calculate the loss rate inside the chamber and behind the mask for all non-ventilation processes. Here, *C* is the concentration of the species inside the chamber or behind the mask, *t* is time, and λ is the decay rate of the species.
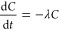
1A mass balance model was then used to understand how the primary disinfectant and its byproducts behaved following the injection of the disinfectant and hours after injection ended. We assessed when the mask or the chamber behaved as a source or a sink during the experiments. Equations 2 – 5 show the mass balance equations utilized for these calculations. Details on the model equations are presented elsewhere for bleach disinfectants.^32^ In summary, *C*_C_ is the species concentration in the chamber. *C*_out_ is the concentration of the species outside the chamber. *C*_M_ is the species concentration inside the mask. *Q̇*_C_ is the volumetric flow rate through the chamber. *Q̇*_B_ is the manikin inhalation rate of 6 L min^–1^, and *t* is time. The mask volume and the chamber volume are defined as two separate terms, *V*_M_ and *V*_C,_ respectively, with *V*_M_ assumed to be 6 L, which is the same as the volume of a healthy adult lung.^37^ Although it is possible to utilize other choices of mask volume, calculations varying the mask volume showed only impacts on the order of magnitude of the calculated net source–sink term and no impact on the qualitative trend. NS_C_ and NS_M_ are the net source–sink terms for the chamber and the mask, respectively, in units of μg h^–1^, and these represent any variation not accounted for by ventilation through the chamber or mask, respectively. A negative net source–sink term indicates a sink, while a positive term indicates a source.
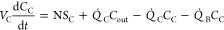
2
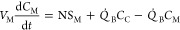
3
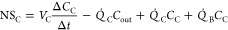
4
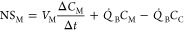
5Net source–sink terms were calculated for the bulk family sum of C_*X*_H_*Y*_O_*Z*_ and C_*X*_H_*Y*_O_*Z*_N_1–2_. To calculate the net source–sink in mass change per hour (μg h^–1^), we utilized estimated average molecular weights of 56 g/mol for C_*X*_H_*Y*_O_*Z*_ and 97 g/mol for C_*X*_H_*Y*_O_*Z*_N_1–2_, calculated from the most abundant compounds in each compound family during the disinfectant injection period from 0 to 3 h, as shown in Table 2.

**Table 2:** Relative Abundance of the Most Abundant C_*X*_H_*Y*_O_*Z*_ and C_*X*_H_*Y*_O_*Z*_N_1–2_ Compounds Inside the Chamber to Their Respective Families from Experiment 1, Calculated from 0 to 3 h

C_*X*_H_*Y*_O_*Z*_	relative abundance to total C_*X*_H_*Y*_O_*Z*_ (%)	C_*X*_H_*Y*_O_*Z*_N_1–2_	relative abundance to total C_*X*_H_*Y*_O_*Z*_N_1–2_ (%)
C_2_H_2_O	16	C_2_H_6_O_3_N_2_	20
C_2_H_4_O	12	C_3_H_5_O_2_N	21
C_2_H_4_O_2_	13	C_6_H_11_ON	13
C_3_H_4_O	3	C_5_H_11_ON	10
C_3_H_4_O_2_	2	C_2_H_5_O_2_N	9
C_3_H_6_O	32	C_4_H_5_O_2_N	9
C_3_H_6_O_2_	3	C_4_H_7_ON	8
C_4_H_6_O_2_	2		
C_4_H_8_O	5		
total	88	total	90
others	12	others	10

## 3 Results and Discussions

### 3.1 Disinfection with Hydrogen Peroxide Emits Oxygen and Nitrogen-Containing Organic Compounds in Addition to the Primary Disinfectant (H_2_O_2_)

**Figure 1:**
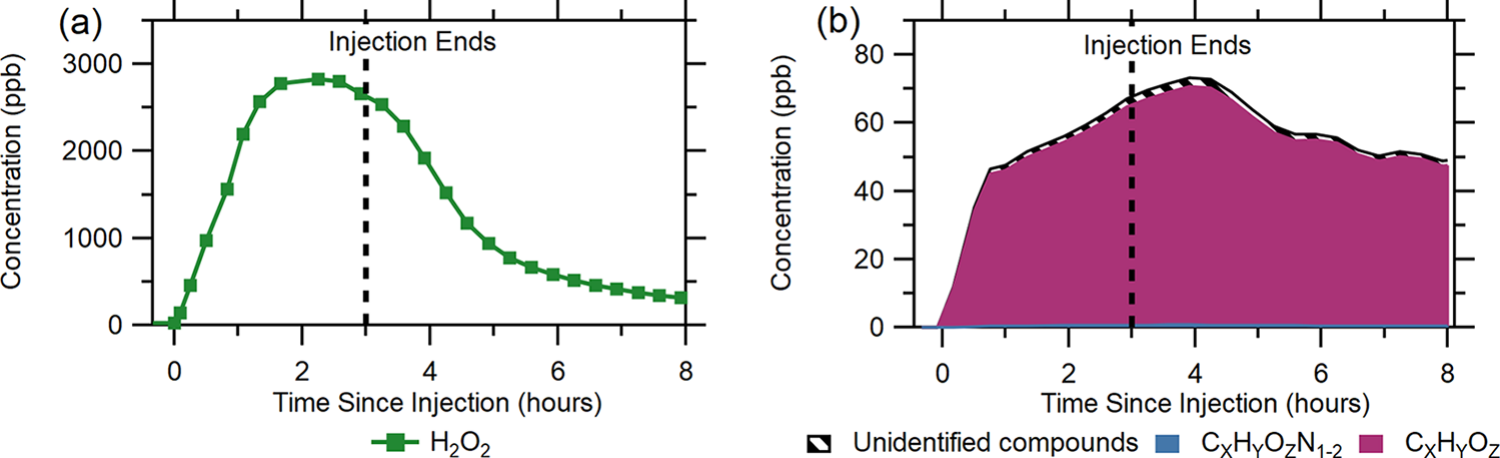
Inside chamber time series of (a) H_2_O_2_ concentrations and (b) bulk concentrations of the byproduct chemical families C_*X*_H_*Y*_O_*Z*_ and C_*X*_H_*Y*_O_*Z*_N_1–2_ from experiment 1.

Elevated H_2_O_2_ concentrations in the chamber were observed in each experiment after injection began. In experiment 1 (Figure 1a), H_2_O_2_ increased gradually for about 2.5 h during the injection period and peaked approximately when injection ended. H_2_O_2_ then decayed at a rate of 0.49 h^–1^, which is much slower than the chamber air change rate of 2.3 h^–1^, suggesting some source of H_2_O_2_ in the chamber, likely desorption from the chamber surfaces after disinfection had ended. Concentrations in the chamber remained elevated at about 200 ppb above background 4 h after injection ended (7 h after the start of the disinfection event); however, they remained below the Occupational Safety and Health Administration’s (OSHA) permissible exposure limit (PEL) of 1 ppm H_2_O_2_ for an 8 h work shift and were only elevated above 1 ppm for about 4 h during the experiment.^38^ For context, the background concentration of hydrogen peroxide in the chamber prior to the start of the disinfection experiments was <1 ppb. This concentration was similar to typical H_2_O_2_ concentrations of <1 ppb measured outdoors and in a simulated residential room.^21,39,40^ Background concentrations between experiments were 18 ± 6 ppb, and all hydrogen peroxide data shown in the text were background subtracted (the background concentrations for each experiment are shown in Table S1).

In addition to hydrogen peroxide, we measured multiple organic compounds with elevated signals following the injection of H_2_O_2_: 85 ions were observed to increase in concentration following H_2_O_2_ injection. Some of these ions may be fragmentation products of larger molecules or water clusters. Figure 1b shows a stacked time series of the sum of the chamber concentrations of the compound families C_*X*_H_*Y*_O_*Z*_ and C_*X*_H_*Y*_O_*Z*_N_1–2_, and unidentified species (compounds for which no chemical formula was identified) during the disinfection event. The two compound families shown are oxygenated organic compounds (C_*X*_H_*Y*_O_*Z*_ family) and some nitrogen-containing organic compounds (C_*X*_H_*Y*_O_*Z*_N_1–2_ family). Concentration increases in VOCs in the C_*X*_H_*Y*_O_*Z*_ and C_*X*_H_*Y*_O_*Z*_N_1–2_ families during each experiment may occur for several reasons. Compounds may be directly emitted from the disinfectant solution as droplets are generated and evaporate. Additionally, compounds may also be emitted indirectly, potentially forming through condensed-phase reactions that occur following H_2_O_2_ surface uptake (either direct adsorption or dissolution into surface water) or being displaced from surfaces by the adsorption of other species. At the RH values typical of these experiments, we expect that surface water layers are present on all chamber surfaces throughout each experiment; so, we suspect that surface displacement of VOCs by water would not be a major pathway by which VOCs increase in concentration. Out of the observed 85 ions, 49 of them were from the C_*X*_H_*Y*_O_*Z*_ family, 16 were from the C_*X*_H_*Y*_O_*Z*_N_1–2_ family, and 20 were unidentified. Of these, the C_*X*_H_*Y*_O_*Z*_ family had by far the highest overall concentrations at approximately 94% of the total signal. Table 2 shows the relative abundance of the compounds to their respective families, which make up ∼90% of the C_*X*_H_*Y*_O_*Z*_ and C_*X*_H_*Y*_O_*Z*_N_1–2_ families. Compounds with chemical formulas of C_2_H_4_O, C_3_H_4_O, and C_3_H_6_O are consistent with the observation of mainly C_1_–C_4_ compounds in other studies of hydrogen peroxide vapor disinfection.^21,24^ Some compounds listed in Table 2 (such as C_2_H_2_O and C_3_H_4_O) are likely fragmentation products of larger, potentially unidentified, molecules.

Similar to H_2_O_2_, the concentrations of C_*X*_H_*Y*_O_*Z*_ and C_*X*_H_*Y*_O_*Z*_N_1–2_ inside the chamber also increased following injection of the disinfectant, as shown in Figure 1b. Concentrations of the byproducts increased from the start of injection up to 1 h after injection ended, indicating that both a direct emission source and an indirect emission source existed in the chamber. The direct emissions here are compounds which originate from the H_2_O_2_ solution, and the indirect emissions are from all other sources, which may include the following: desorption of deposited compounds from surfaces, chemicals displaced from surfaces by water sorbed onto surfaces,^41^ and surface reactions between deposited H_2_O_2_, surface materials, and adsorbed semi-volatile organic compounds (SVOCs) present on the chamber walls or tables in the chamber. After concentrations peaked, they began to decay steadily; however, they remained elevated up to 4 h after injection ended at a bulk concentration of about 45 ppb. Health impacts of the byproducts are not discussed because our instruments identify molecular formulae, and not chemical identities.

### 3.2 Concentrations of Emitted Compounds Remain Elevated Behind Mask and Inside Chamber after Disinfection

**Figure 2:**
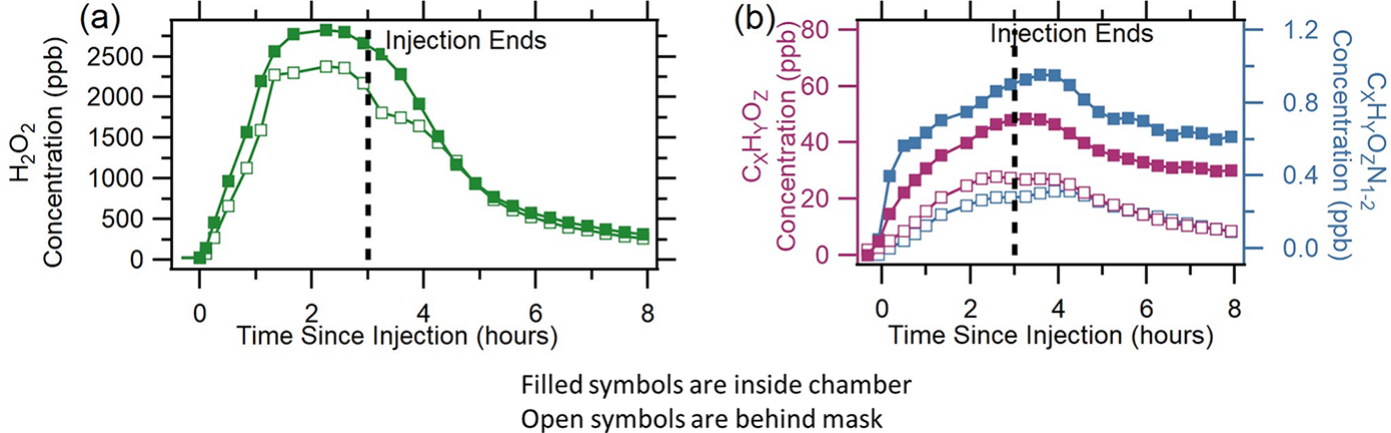
Time series of concentrations behind mask and inside chamber from dry KN95 mask experiment 1 for (a) H_2_O_2_ and (b) the C_*X*_H_*Y*_O_*Z*_ and C_*X*_H_*Y*_O_*Z*_N_1–2_ compound families.

Concentrations of H_2_O_2_ behind the mask remained overall lower than those inside the chamber during and immediately following the injection period, and they also increased at a slower rate (Figures 2a and S2). In the dry KN95 mask experiment (experiment 1), concentrations behind the mask remained lower or at a level similar to that of concentrations inside the chamber (Figure 2a). Humidified and dry surgical mask experiments (experiments 2 and 5, Figure S2a) showed behind-mask concentrations of H_2_O_2_ that decayed more slowly than the chamber concentrations and became higher than the concentrations inside the chamber about 4 h after injection ended. The higher concentrations at the end of the experiments and differences in the decay rate of H_2_O_2_ behind the mask (Table 1) are attributed to the re-emission of adsorbed H_2_O_2_ from the mask surface. We speculate that concentrations behind the mask became higher in the surgical mask experiments than the KN95 mask experiments because of differences in mask type, which may result in higher adsorption of H_2_O_2_ on surgical masks than KN95 masks. H_2_O_2_ data were not available for experiments 3 and 4. Similar to those inside the chamber, H_2_O_2_ concentrations behind the mask also remained elevated above background levels at about 200 ppb in experiments 1 and 5 and at about 50 ppb in experiment 2. These elevated concentration levels are also below the OSHA permissible exposure limit (PEL) of 1 ppm of H_2_O_2_ for an 8 h work shift.^38^

The C_*X*_H_*Y*_O_*Z*_ and C_*X*_H_*Y*_O_*Z*_N_1–2_ compound families showed growth trends similar to that of H_2_O_2_, where concentrations in the chamber increased more rapidly than those behind the mask. The concentrations of C_*X*_H_*Y*_O_*Z*_ and C_*X*_H_*Y*_O_*Z*_N_1–2_ inside the chamber also remained elevated over those behind the mask during the injection period. Following injection, C_*X*_H_*Y*_O_*Z*_ concentrations in experiment 3 (Figure S3b) were elevated behind the mask longer than inside the chamber. The elevated concentrations behind the mask indicate that if the same mask is worn during and following a disinfection event, the mask may become a source of higher concentration exposure than air within the disinfected space (depending on the air change rate). In a humidified KN95 experiment, shown in Figure S3a, concentrations behind the mask remained lower than concentrations inside the chamber; however, these concentrations were still elevated above background levels, indicating that despite the lower concentrations behind the mask, some level of exposure is still possible. Concentrations reported in the text are background subtracted, and background concentrations of the compound families from each experiment are reported in the SI (Table S1). We emphasize that surgical, N95, and KN95 masks are designed for protection against the inhalation of aerosols and not gas-phase pollutants.^16−18,42^ We have no data on the byproducts during the surgical mask experiments due to the unavailability of the Vocus during those experiments.

**Figure 3:**
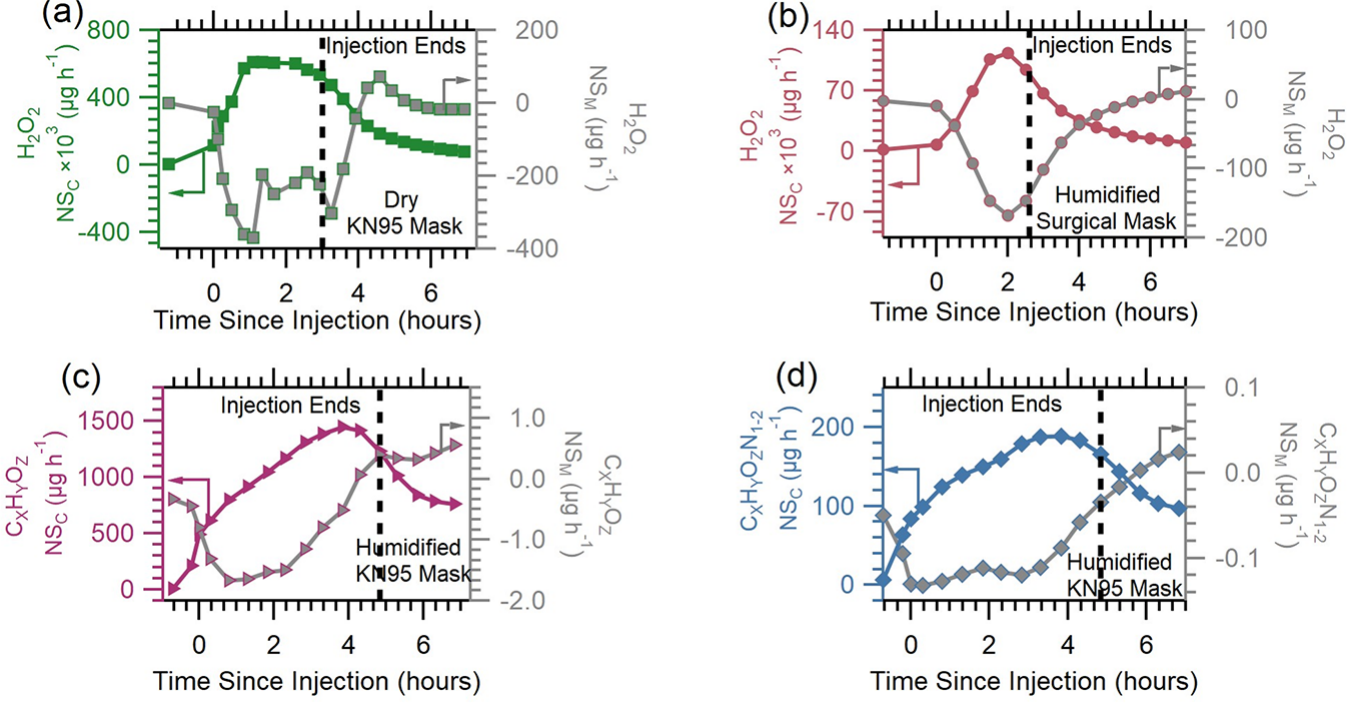
Net source–sink profiles of (a) H_2_O_2_ in dry KN95 mask experiment 1, (b) H_2_O_2_ in humidified surgical mask experiment 2, (c) the C_*X*_H_*Y*_O_*Z*_ family in humidified KN95 mask experiment 3, and (d) the C_*X*_H_*Y*_O_*Z*_N_1–2_ family in humidified KN95 mask experiment 3. Arrows show the axis corresponding to the trace.

To gain more insight into the chamber and mask emission source–sink dynamics, we calculated net source–sink parameters for the chamber (NS_C_) and the mask (NS_M_). A positive net source–sink (NS) value indicates a source, while a negative net source–sink value indicate a sink. In Figure 3a,b, the NS_C_ for H_2_O_2_ is positive (NS_C_ > 0) at the start of injection and increases to a stable level for the duration of the injection before decaying toward 0 following injection. This shows that, as expected, the chamber became a source of H_2_O_2_ when the disinfectant was injected and remained a source after injection ended, presumably due to some H_2_O_2_ desorbing from the surfaces in the chamber. This pattern is seen with the dry KN95 mask, the humidified surgical mask (experiments 1 and 2, panels a and b of Figure 3, respectively), and the dry surgical mask (experiment 5, Figure S4). In contrast, the NS_M_ parameter decreased during injection to a negative value, indicating that concentrations behind the mask were lower than those in the chamber and that a sink was present across the mask. After injection ended, NS_M_ increased until it became positive 4 h after the end of injection, indicating the mask became a source of H_2_O_2_. One possible reason for this is adsorption of H_2_O_2_ onto the mask surface and gradual desorption of this compound over time. This would be consistent with prior work by Kumkrong et al.,^25^ in which elevated concentrations of H_2_O_2_ over a 0.5–5 h period were observed after N95 masks had been disinfected for reuse, as well as in similar work on bleach emissions and their interactions with mask surfaces.^32^ The chamber acts as a source of the detected compounds, either directly from injection or indirectly from reactive formation or desorption, possibly involving some water layers on surfaces.^41^ The mask surface initially acts as a sink, where compounds are adsorbed or deposited, and subsequently as a source when adsorbed compounds are desorbed later in the experiment.^22,43−45^

The NS_C_ parameter for both C_*X*_H_*Y*_O_*Z*_ and C_*X*_H_*Y*_O_*Z*_N_1–2_ (Figure 3c,d) increased during injection from negative values to positive values and then decreased while remaining positive for 2 h after the end of injection. This is consistent with the injection of H_2_O_2_ not acting as a primary source for C_*X*_H_*Y*_O_*Z*_ and C_*X*_H_*Y*_O_*Z*_N_1–2_ compounds. Instead, these compounds gradually form, desorb, or are displaced from chamber surfaces. The NS_M_ term was slightly negative prior to injection and initially decreased for approximately 1 h before finally increasing and eventually becoming positive. These observations imply that the chamber started out as a source and remained one for the duration of the experiment, while the mask was initially a sink before becoming a source hours after injection ended. The consistent source of C_*X*_H_*Y*_O_*Z*_ and C_*X*_H_*Y*_O_*Z*_N_1–2_ within the chamber could result from continuous reactive formation, the emission of VOCs within the chamber, or the displacement of VOCs present on surfaces by water. Similar compounds to those which make up the C_*X*_H_*Y*_O_*Z*_ family have been noted in previous work with hydrogen peroxide as the disinfectant.^16,18^ Although the mask acted primarily as a sink during the experiment, toward the end it eventually became a source of hydrogen peroxide, as seen in the scenarios highlighted here. Similarly, the mask also acted as a sink for byproducts for the majority of the experiment and only became a source in some instances. This implies that while these masks when worn during disinfection with H_2_O_2_ initially reduce the concentration the user is exposed to, over the course of time they can become a source of higher exposure. The proximity of those concentrations to the breathing zone and skin surface of the user is a further concern. Such exposures can be mitigated by replacing masks worn during high chemical concentration exposures, for instance during disinfection or while handling other chemicals, once concentrations are lower.

### 3.3 Effects of Mask Humidification on Behind-Mask Concentrations

The impacts of humidifying the mask surface on behind-mask concentrations (i.e., exposure) were tested by conducting otherwise identical experiments with dry masks and masks that were humidified prior to H_2_O_2_ injection. Peak concentrations of H_2_O_2_ (Table 1) recorded in experiments 1 (dry KN95 mask), 2 (humidified surgical mask), and 5 (dry surgical mask) were lower behind the mask; however, pre-humidifying the mask showed no clear impact. To quantify these effects, we calculated the decay rates of H_2_O_2_ behind the mask and inside the chamber (Table 1) for the period after injection ended to understand how H_2_O_2_ was lost through reactions or surface deposition. H_2_O_2_ decay rates within the chamber were nearly identical in the dry and humidified mask experiments, ranging from 0.48–0.50 h^–1^. Similar decay rates were expected inside the chamber across varying experimental conditions because the chamber AER (Table 1) and RH were kept constant across all experiments and mask pre-humidification does not substantially affect chamber RH. The inside-chamber H_2_O_2_ decay rates were lower than the AER of 2.3 h^–1^ measured during the experiments. These slow decay rates inside the chamber indicate a source in the chamber, potentially re-emission of H_2_O_2_ from surface deposition or from water layers on surfaces.^41^ Behind the mask, we observed a H_2_O_2_ decay rate of 0.47 h^–1^ in the dry KN95 mask experiment (experiment 1), a decay rate of 0.15 h^–1^ in the dry surgical mask experiment (experiment 5), and a decay rate of 0.17 h^–1^ in the humidified surgical mask experiment (experiment 2). From this, the decay rates of hydrogen peroxide behind the surgical masks were observed to be lower than the decay rates of hydrogen peroxide behind the KN95 mask. The similar decay rates between the dry and humidified surgical mask experiments suggest that the lower decay rates are because of the mask type and not humidification. Lower decay rates in the surgical masks indicate that H_2_O_2_ is being lost at a slower rate behind the surgical mask than behind the KN95 mask. This may be a result of faster desorption of adsorbed H_2_O_2_ from the surgical mask surface, the lower capacity of the surgical mask as a reservoir via adsorption, or differences in the mask fit. In addition, the lower decay rates observed in both surgical mask experiments are consistent with positive NS_M_ values, showing that the mask becomes a source of H_2_O_2_ to the gas phase, as shown in Figures 3b and S4.

**Figure 4:**
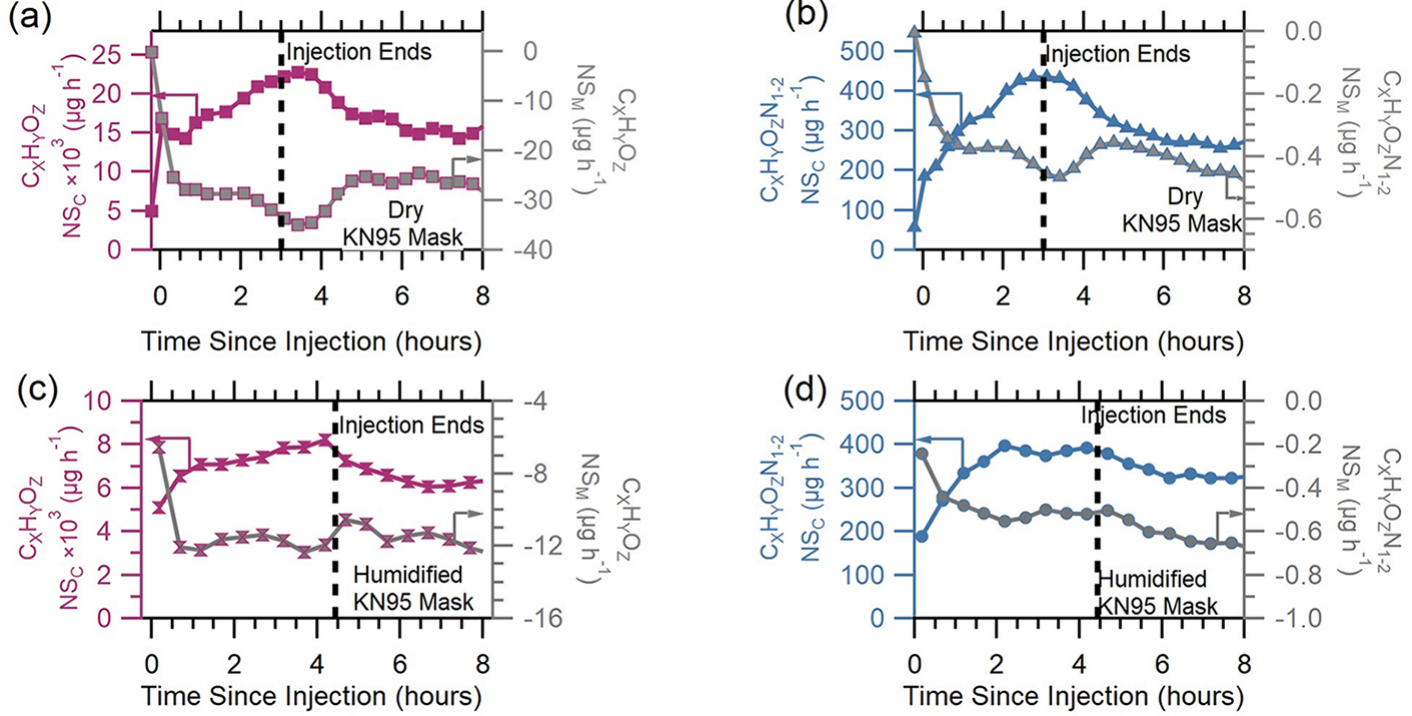
Net source–sink of byproduct families (a) C_*X*_H_*Y*_O_*Z*_ from dry KN95 mask experiment 1, (b) C_*X*_H_*Y*_O_*Z*_N_1–2_ from dry KN95 mask experiment 1, (c) C_*X*_H_*Y*_O_*Z*_ from humidified KN95 mask experiment 4, and (d) C_*X*_H_*Y*_O_*Z*_N_1–2_ from humidified KN95 mask experiment 4. Arrows show the axis corresponding to the trace.

Finally, we examined the effects of mask humidification on the byproducts from disinfection with hydrogen peroxide. Figure 4 shows the net source–sink plots from dry KN95 experiment 1 and humidified KN95 experiment 4. In all plots for both byproduct families, the NS_C_ parameter increased during injection until it peaked, after which it began to decrease but remained positive for the duration of the experiment. The implication of this positive NS_C_ value is that the chamber remained a source of byproducts during disinfection and hours after disinfection ended from continued indirect emissions of the byproducts. However, the NS_M_ value was negative throughout the disinfection experiment, indicating that in these experiments, the mask did not become a source of the byproducts at any point during the disinfection process. Observations made here also show no clear effects of mask humidification.

## 4 Conclusions

In this work, we simulated hydrogen peroxide disinfection of an indoor space to evaluate the byproducts emitted as a result of the use of hydrogen peroxide for disinfection and the effect of wearing facial coverings on personal exposure to the primary disinfectant and byproducts. We identified 85 ion signatures (excluding H_2_O_2_) that increased in association with the disinfection event that were primarily oxygen-containing organics (C_*X*_H_*Y*_O_*Z*_) but also included smaller concentrations of nitrogen-containing organics (C_*X*_H_*Y*_O_*Z*_N_1–2_). Inside the chamber, concentrations of hydrogen peroxide and the associated byproducts persisted at elevated concentrations for 2–4 h after injection ended, indicating a persistent source, potentially from the surfaces in the chamber. Hydrogen peroxide concentrations remained below the OSHA permissible exposure limit (PEL) of 1 ppm of H_2_O_2_ for an 8 h work shift.^38^ Concentrations of hydrogen peroxide and some byproducts behind the mask remained elevated above those in the chamber 2–4 h after injection ended, indicating an emission source behind the mask, which we suspect is the mask surface. A net source–sink analysis confirmed that surgical masks eventually become a source of H_2_O_2_ while KN95 masks remain a sink of H_2_O_2_. The KN95 mask became a byproduct emission source in some experiments a few hours after injection ended; however, we have no data on the byproducts that formed during the surgical mask experiments. Finally, decay rates showed that hydrogen peroxide was lost at a slower rate behind the mask than inside the chamber, pointing to the possibility of extended exposure to higher hydrogen peroxide concentrations from mask emissions relative to concentrations within a disinfected indoor space. Mask humidification did not impact the exposure to hydrogen peroxide or byproducts. However, mask type impacted exposure by as much as a factor of 2. With the mask surface being a potential source of emissions hours after disinfectant injection ended and given the typical proximity to the breathing zone, we recommend that mask users change their mask following exposure to high concentrations of disinfectants as well as other chemicals to reduce potential inhalation following the re-emission of adsorbed species.

## Data Availability

Data related to the figures reported in this manuscript are available in the Texas Data Repository: https://doi.org/10.18738/T8/NYDPI9. Additional data including raw data are available upon request.

## Supporting Information Available

Description of the mask humidification process, hydrogen peroxide calibrations, chamber background concentrations, additional hydrogen peroxide time series behind mask and inside chamber, bulk composition time series inside the chamber, net source–sink profiles, and chamber relative humidity time series (PDF)
