# Supplementary material for: Emissions from Hydrogen Peroxide Disinfection and Their Interaction with Mask Surfaces
Source: ACS Eng Au. 2024 Jan 9;4(2):204–12. doi: 10.1021/acsengineeringau.3c00036 (PMC11027093; doi:10.1021/acsengineeringau.3c00036)
Supplement: Supplementary file 1 — eg3c00036_si_001.pdf [file eg3c00036_si_001.pdf]

Supplementary Information: Emissions from hydrogen peroxide disinfection and their interaction with mask surfaces

Pearl Abue[1], Nirvan Bhattacharyya[1], Mengjia Tang[2], Leif G. Jahn[1], Daniel Blomdahl[2], David T. Allen[1], Richard L. Corsi[3], Atila Novoselac[2], Pawel K. Mistzal[2], Lea Hildebrandt Ruiz[1, *].

[1]McKetta Department of Chemical Engineering, The University of Texas at Austin, Austin, TX, 78712, USA

[2]Department of Civil, Architectural, and Environmental Engineering, The University of Texas at Austin, Austin, TX, 78712, USA

[3]College of Engineering, University of California at Davis, Davis, CA, 95616, USA

*Corresponding author: lhr@che.utexas.edu

SI. 1: Humidification of masks

KN95 and surgical masks used in the experiments were removed from their packaging and then immediately used either dry or humidified for the experiments. Air sourced from the laboratory, passed through a HEPA filter and then through a bubbler containing deionized water was exhaled on to the mask surface for about 60 minutes prior to the start of experiments. The humidified air (RH > 95 %, T = 37 °C) was exhaled onto the mask surface at 12 breaths min^{-1} corresponding to an exhalation rate of 6 L min^{-1}. KN95 masks, after humidification, weighed an average of 1 ± 0.2 g more than dry masks indicating that about 1 g of water had been adsorbed onto the surface of the masks. We do not have data on the weight of surgical masks following humidification.

SI. 2: Hydrogen peroxide calibrations

Hydrogen peroxide calibrations on the CIMS were conducted by injecting H_2O_2 into a 10 m^3 Teflon environmental chamber by bubbling clean, dry air at 2 LPM through an aqueous 30% hydrogen peroxide solution. A multipoint calibration was performed by injecting different volumes of hydrogen peroxide into the chamber to obtain different concentrations. We utilize a photometric ozone monitor which detects H_2O_2 as ozone to estimate concentrations of H_2O_2 injected into the chamber. More specifically, we estimate H_2O_2 concentrations by calculating the ratio of the absorption cross sections of H_2O_2 at 254nm to ozone at 254 nm[1] multiplied by the measured "ozone" concentration.

SI 3: Compound family background concentrations

The concentrations reported in the text were all background subtracted. We report the background concentrations from each experiment in Table S1.

Table S1: Background concentrations inside chamber and behind mask

#	Mask	Instrumentation	Inside Chamber H_2O_2 (ppb)	Behind Mask H_2O_2 (ppb)	Inside Chamber $C_xH_yO_z$ (ppb)	Behind Mask $C_xH_yO_z$ (ppb)	Inside Chamber $C_xH_yO_zN_{1-2}$ (ppb)	Behind Mask $C_xH_yO_zN_{1-2}$ (ppb)
1	KN95	CIMS, Vocus	20	15	71	25	1	1
2	Surgical	CIMS	11	5	-	-	-	-
3	KN95	Vocus	-	-	39	16	1	1
4	KN95	Vocus	-	-	5	26	1	2
5	Surgical	CIMS	23	16	-	-	-	-

SI. 4: Bulk composition timeseries of compound families and percentage contribution of each family

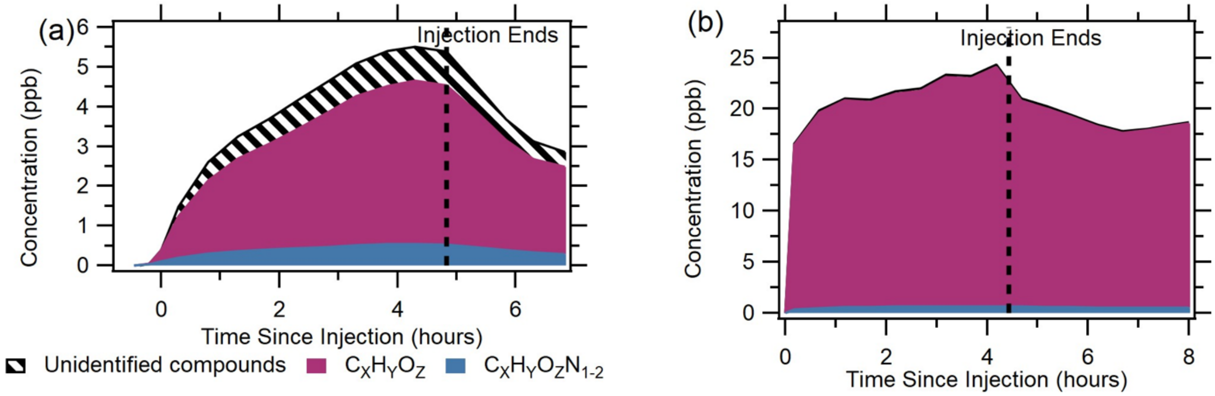

Figure S1: Bulk concentrations of byproduct chemical families, $C_XH_YO_Z$ and $CXH_YO_ZN_{1-2}$ **a.** Humidified KN95 mask from experiment 3 **b.** Humidified KN95 mask experiment 4

SI. 5: Inside the chamber and behind the mask timeseries

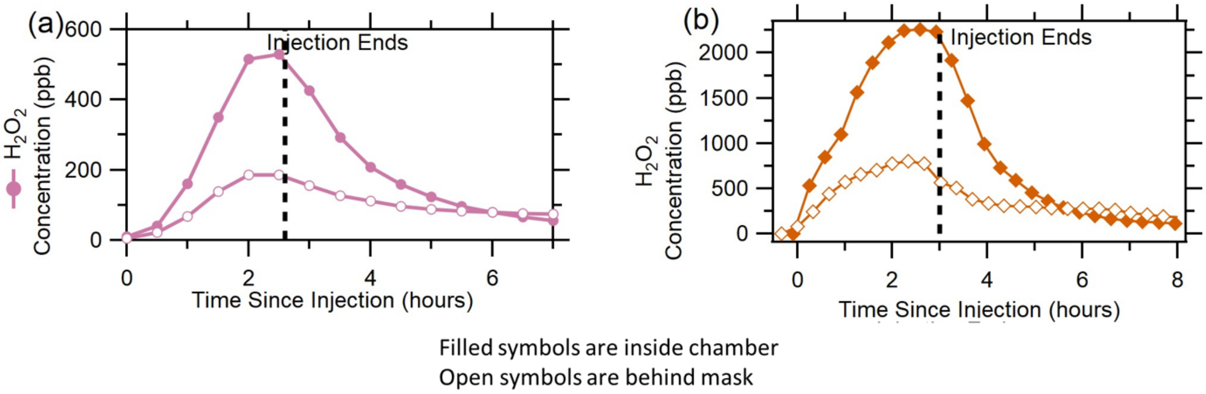

Figure S2: Timeseries of behind mask and inside chamber concentrations for **a.** H_2O_2 from experiment 2-Humidified surgical mask experiment **b.** H_2O_2 from experiment 5 – dry surgical mask experiment.

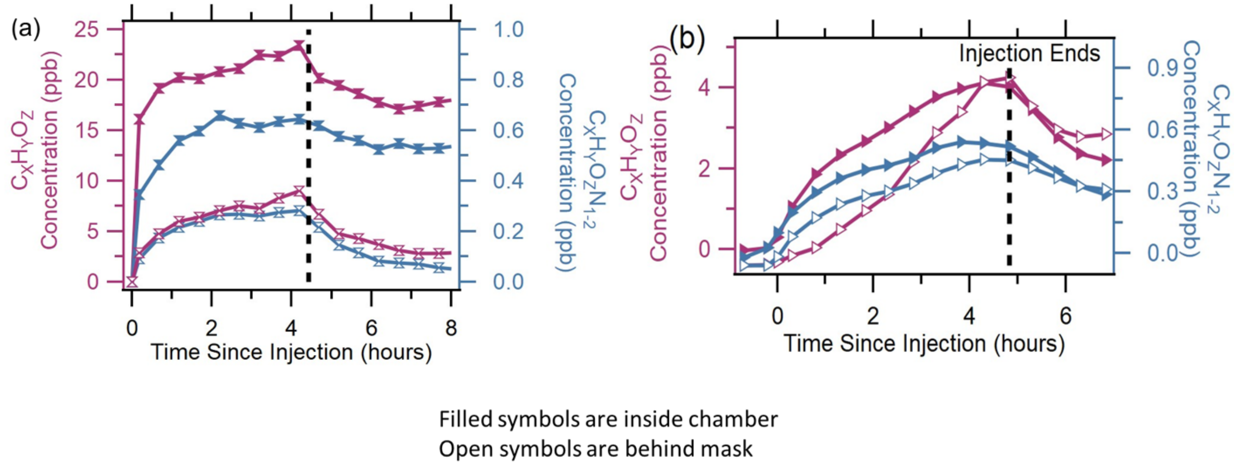

Figure S3: Timeseries of behind mask and inside chamber concentrations for compound families from **a.** humidified KN95 mask experiment 4 **b.** humidified KN95 experiment 3.

SI. 6: Net source-sink profiles

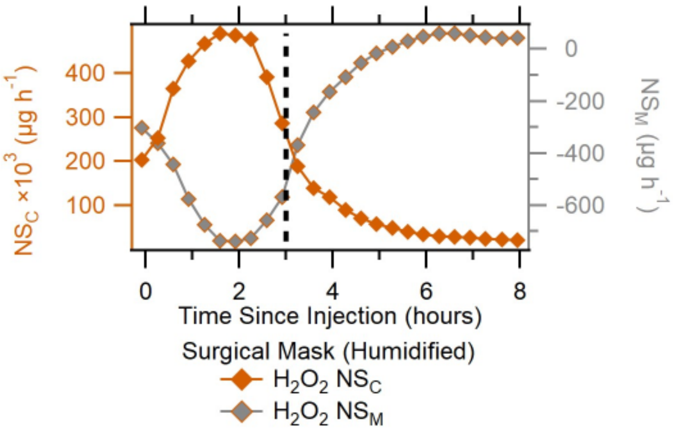

Figure S4: Net source-sink of hydrogen peroxide from experiment 5 (dry surgical mask)

SI 4: Chamber relative humidity

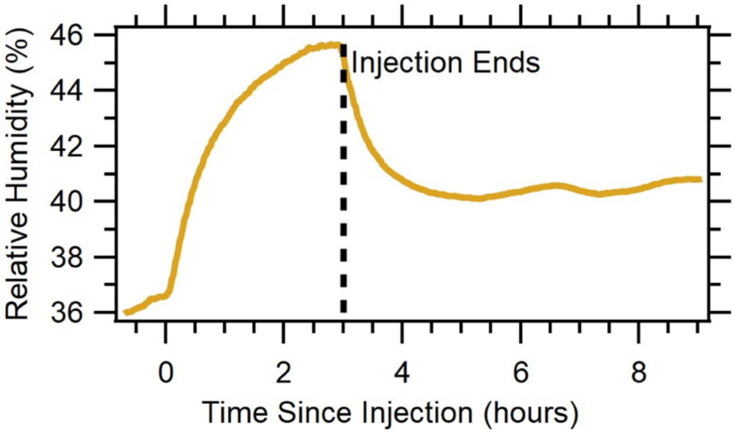

Figure S5: Timeseries of relative humidity in the environmental chamber during experiment 5

References

1. J. B. Burkholder, S. P. Sander, J. Abbatt, J. R. Barker, C. Cappa, J. D. Crounse, T. S. Dibble, R. E. Huie, C. E. Kolb, M. J. Kurylo, V. L. Orkin, C. J. Percival, D. M. Wilmouth, and P. H. Wine "Chemical Kinetics and Photochemical Data for Use in Atmospheric Studies, Evaluation No. 19," JPL Publication 19-5, Jet Propulsion Laboratory, Pasadena, 2019 http://jpldataeval.jpl.nasa.gov.
